# Chronotype and Atherogenic–Adiposity Indices in Adults Without Previously Diagnosed Chronic Disease: A Sex-Aware Cross-Sectional Study

**DOI:** 10.3390/medicina62081472

**Published:** 2026-07-29

**Authors:** Kemal Ozan Lule, Serpil Şahin, Nezihe Otay Lule, Mert Deniz Savcilioglu, Hamit Yildiz

**Affiliations:** 1 Internal Medicine Department, Faculty of Medicine, Gaziantep University, 27310 Gaziantep, Türkiye; serpilsahin.6327@gmail.com (S.Ş.); drhyildiz@hotmail.com (H.Y.); 2 Nutrition and Dietetics Department, Faculty of Health Sciences, Gaziantep University, 27310 Gaziantep, Türkiye; otaynezihe@hotmail.com; 3 Cardiology Department, Faculty of Medicine, Gaziantep University, 27310 Gaziantep, Türkiye; mdsavcilioglu@gmail.com

**Keywords:** chronotype, sleep quality, visceral adiposity index, atherogenic index of plasma, sex differences, cardiometabolic risk, circadian rhythm

## Abstract

*Background and Objectives*: Chronotype and sleep quality are related but non-identical dimensions of sleep health. We examined whether later chronotype is associated with subclinical visceral adiposity and atherogenic dyslipidaemia independently of perceived sleep quality, and whether this association differs by sex, in adults without previously diagnosed chronic disease. *Materials and Methods*: This single-centre cross-sectional study included 282 adults without previously diagnosed chronic disease or regular medication use. Chronotype was assessed with the Morningness–Eveningness Questionnaire (MEQ) and sleep quality with the Pittsburgh Sleep Quality Index (PSQI). Composite cardiometabolic indices were calculated from routine anthropometric and biochemical data. Primary dependent variables were log-transformed Visceral Adiposity Index (log[VAI]) and Atherogenic Index of Plasma (AIP). Multivariable linear models used heteroscedasticity-consistent type 3 (HC3) robust standard errors and adjusted for age, sex, education, physical activity, and PSQI; body mass index (BMI) was additionally included in the AIP model. Sex × MEQ interactions, false-discovery-rate (FDR) correction, and glycaemic sensitivity analyses were performed. *Results*: Median age was 28.0 years; 50.4% were women, 25.9% were evening type, and 51.8% had poor sleep quality. Higher MEQ score (greater morningness) was independently associated with lower log(VAI) (B = −0.0096; 95% confidence interval (CI), −0.0159 to −0.0033; β = −0.190; *p* = 0.003) and AIP (B = −0.0035; 95% CI, −0.0063 to −0.0008; β = −0.160; *p* = 0.011), whereas PSQI was not independently associated with either outcome. Sex × MEQ interactions were nominally significant and estimates were stronger in women, but interaction terms did not survive FDR correction. Associations persisted after excluding diabetes-range glycaemia but attenuated in strictly normoglycaemic participants. *Conclusions*: Later chronotype was associated with a less favourable subclinical adiposity–atherogenic profile independently of perceived sleep quality. Findings are cross-sectional, and the sex-specific pattern is hypothesis-generating. Prospective studies with objective circadian, sleep, dietary-timing, behavioural, and hormonal measures are required.

## 1. Introduction

Circadian rhythmicity is increasingly recognised as a behavioural and biological determinant of cardiometabolic health [1,2]. Chronotype, commonly defined as an individual preference for earlier or later timing of sleep and daily activity, has been associated with differences in dietary behaviour, physical activity, sleep timing, adiposity, glucose metabolism, and lipid profile [3,4,5]. Evening chronotype, in particular, has been linked to less favourable cardiometabolic patterns in non-shift-working populations [6,7], although such associations are often difficult to separate from sleep quality, lifestyle behaviours, and pre-existing disease burden.

Sleep quality is related to chronotype but is not equivalent to it. Poor sleep health, including disturbances in sleep quality and sleep duration, has been associated with obesity and adverse cardiometabolic profiles [8,9], and is captured by validated instruments such as the Pittsburgh Sleep Quality Index (PSQI) [10,11]; however, it may reflect fragmented or insufficient sleep, psychological distress, or environmental factors, whereas chronotype reflects phase preference and the timing of behaviour relative to social demands [12]. Entering both constructs into the same model is therefore important, because otherwise the apparent metabolic contribution of either may be overestimated.

Sex may further modify chronotype-related cardiometabolic expression, as several studies have reported sex-related variation in chronotype, adiposity, lipid profile, and metabolic-risk associations [13,14,15,16,17]. Many such analyses, however, rely on stratified description rather than formal interaction testing, so a sex-aware analytical framework is needed to determine whether the same chronotype score carries similar metabolic meaning in women and men.

Early cardiometabolic risk is not always captured adequately by body mass index (BMI) or by isolated lipid concentrations [18]. Composite indices—including the Visceral Adiposity Index (VAI), Lipid Accumulation Product (LAP), Atherogenic Index of Plasma (AIP), Body Roundness Index (BRI), waist-to-height ratio (WHtR), and triglyceride–glucose-related waist indices—integrate anthropometric and biochemical information and may better reflect subclinical visceral adiposity, lipid accumulation, and atherogenic dyslipidaemia [19,20,21,22,23,24]. These indices are attractive in outpatient settings because they are derived from routinely available measurements.

Although chronotype has previously been linked to obesity, lipid profile, and metabolic syndrome, several gaps remain. First, chronotype and sleep quality are often examined separately rather than in mutually adjusted models, making it difficult to determine whether later chronotype carries cardiometabolic information beyond perceived sleep quality. Second, many cohorts include participants with established chronic disease or medication use, which may obscure earlier phenotypic signals that precede overt diagnosis or treatment. Third, sex-specific effects are frequently inferred from stratified comparisons rather than tested formally using interaction terms. Finally, few studies have compared multiple routinely derived adiposity and atherogenic indices within the same early-phenotyping framework. Accordingly, the present study had three prespecified aims: (1) to examine whether chronotype and sleep quality were independently associated with subclinical visceral adiposity and atherogenic dyslipidaemia in adults without previously diagnosed chronic disease; (2) to test sex as an effect modifier of the chronotype–cardiometabolic association; and (3) to compare the relative magnitude of chronotype-related associations across routinely derived cardiometabolic indices.

## 2. Materials and Methods

### 2.1. Study Design, Setting, and Participants

This single-centre, cross-sectional observational study was conducted in the General Internal Medicine outpatient clinic of Gaziantep University Sahinbey Research and Practice Hospital. Consecutive adults presenting for outpatient evaluation during the study period were screened for eligibility, and questionnaire, anthropometric, and biochemical data were obtained at enrolment. The flow of participants from screening to analysis is shown in Supplementary Figure S1.

Inclusion criteria were age 18–65 years, absence of previously diagnosed chronic systemic disease, no regular medication use, sufficient Turkish literacy and cognitive capacity to complete questionnaires independently, and written informed consent. The upper age limit reflects the source population, as the general internal medicine outpatient clinic serves adults aged 18–65 years, with older patients evaluated in a separate geriatrics outpatient clinic. Exclusion criteria were a previous diagnosis of type 2 diabetes, hypertension, coronary artery disease, chronic kidney disease, chronic liver disease, chronic lung disease, malignancy, or any systemic chronic condition; regular (daily or near-daily) use of any prescription or over-the-counter medication, including hormonal contraceptives or other hormonal therapies, vitamins, mineral or metabolic supplements, and sleep-related or psychotropic agents; active psychiatric disorder; diagnosed sleep disorder; pregnancy or lactation; shift or night work; transmeridian travel within the preceding four weeks; missing routine biochemical results; and incomplete questionnaire data. Occasional use of a simple analgesic for an acute complaint was permitted.

The final analytical sample comprised 282 adults. Data collection was conducted from 26 March 2026 to 12 June 2026. Physical activity was self-reported and categorised as active if participants reported at least 150 min per week of moderate-intensity aerobic activity, in accordance with the World Health Organization recommendations [25]. The study was approved by the Non-Interventional Clinical Research Ethics Committee of Gaziantep University (Decision No: 2026/221; Date: 25 March 2026) and was conducted in accordance with the Declaration of Helsinki. Written informed consent was obtained from all participants.

### 2.2. Assessment of Chronotype and Sleep Quality

Chronotype was assessed using the validated Turkish adaptation of the 19-item Morningness–Eveningness Questionnaire (MEQ) [26,27]. MEQ scores range from 16 to 86, with higher scores indicating stronger morning preference; participants were classified as evening type (MEQ ≤ 41), intermediate type (42–58), or morning type (≥59). Sleep quality was assessed using the validated Turkish version of the PSQI [10,28]; the global score ranges from 0 to 21, with higher scores indicating poorer sleep, and a score > 5 defined poor sleep quality.

### 2.3. Anthropometric and Biochemical Measurements

Height and weight were measured using calibrated instruments, and BMI was calculated as weight divided by height squared (kg/m^2^). Waist circumference was measured at the midpoint between the lower costal margin and the iliac crest, and WHtR was calculated as waist circumference divided by height. Fasting venous blood samples were analysed for glucose, HbA1c, triglycerides (TG), HDL-cholesterol (HDL-C), LDL-cholesterol, albumin, C-reactive protein, complete blood count parameters, alanine aminotransferase, aspartate aminotransferase, creatinine, urea, thyroid-stimulating hormone, sodium, and potassium using standard automated laboratory methods.

### 2.4. Composite Adiposity and Atherogenic Indices

VAI was calculated using the original sex-specific formulae [19], with waist circumference in cm, BMI in kg/m^2^, and TG and HDL-C in mmol/L: for men, VAI = [waist circumference/(39.68 + 1.88 × BMI)] × (TG/1.03) × (1.31/HDL-C); for women, VAI = [waist circumference/(36.58 + 1.89 × BMI)] × (TG/0.81) × (1.52/HDL-C). LAP was calculated as (waist circumference − 65) × TG for men and (waist circumference − 58) × TG for women, with TG in mmol/L [20]. AIP was calculated as log_10_(TG/HDL-C) with both lipids expressed in mmol/L, in accordance with the original definition by Dobiašová and Frohlich [21]; BRI was calculated using the geometrical model of Thomas et al. [22] as 364.2 − 365.5 × √(1 − [(waist circumference/2π)/(0.5 × height)]^2^), with waist circumference and height expressed in metres, and the triglyceride–glucose index–waist circumference (TyG-WC) as ln[TG (mg/dL) × fasting glucose (mg/dL)/2] × waist circumference (cm) [24].

The Prognostic Nutritional Index (PNI) was not treated as a primary cardiometabolic outcome. When explored descriptively, it was calculated using the standard unit-consistent Onodera formulation [29], albumin (g/L) + 0.005 × total lymphocyte count (/mm^3^), which avoids the unit inflation that occurs if albumin measured in g/L is multiplied by ten.

### 2.5. Statistical Analysis

Sample size was determined a priori for multivariable linear regression using a two-sided significance level of α = 0.05 and 80% statistical power (1 − β = 0.80). Based on a small-to-moderate expected association (r ≈ 0.25), informed by published data on sleep quality and metabolic–anthropometric parameters [30], the minimum required sample size was estimated to be approximately 187 participants. To allow for potential data loss and incomplete records, a minimum enrolment target of 200 participants was planned. The final analytical sample of 282 participants exceeded this threshold.

Continuous variables are presented as mean ± standard deviation (SD) or median (interquartile range, IQR), as appropriate, and categorical variables as *n* (%). The distribution of each continuous variable was assessed before test selection using the Shapiro–Wilk test together with visual inspection of histograms and Q–Q plots, and homogeneity of variance was assessed using Levene’s test. Because formal normality tests are sensitive to minor departures from normality in samples of this size, the choice between parametric and non-parametric tests was based on the combination of these criteria. Variables meeting the assumptions were analysed with parametric tests, and those that did not were analysed with the corresponding non-parametric tests; VAI and LAP were additionally log-transformed for regression modelling because of right-skewed distributions. Between-group comparisons used the independent-samples *t*-test or Mann–Whitney U test for two groups, one-way ANOVA or Kruskal–Wallis test for three groups, and the chi-square test for categorical variables. Multiple comparisons across descriptive cardiometabolic indices were controlled using the Benjamini–Hochberg false-discovery-rate (FDR) procedure.

The primary exposure was MEQ score analysed continuously, with higher values indicating greater morningness; PSQI global score was included as a mutually adjusted sleep-quality exposure. The primary dependent variables were log(VAI) and AIP, and secondary outcomes were log(LAP), BRI, WHtR, and TyG-WC. Multivariable linear regression models were adjusted for age, sex, physical activity status, educational status, and PSQI score. BMI was additionally included in the AIP model because AIP does not mathematically incorporate an anthropometric component; BMI was not included in the VAI/LAP/BRI/WHtR/TyG-WC models to avoid overadjustment for variables embedded in the composite adiposity phenotype. Heteroscedasticity-consistent type 3 (HC3) robust standard errors were used. For each regression coefficient, a 95% confidence interval (CI) was reported. Multicollinearity was assessed using variance inflation factors (VIFs), with VIF > 5 considered indicative of potentially problematic collinearity; observed VIFs did not exceed 1.35 in the main adjusted models or 2.72 in the interaction models. For interaction models, MEQ score was mean-centred before calculating the sex × MEQ product term to reduce non-essential multicollinearity.

Sex × MEQ interaction terms tested effect modification. Because interaction analyses across multiple outcomes increase the probability of false-positive findings, both nominal *p*-values and FDR-adjusted q-values are reported, and sex-stratified models were used to aid interpretation. Participants with prediabetic-range values were retained in the primary analysis because the study aimed to characterise the full pre-diagnostic metabolic spectrum. To isolate their contribution, sensitivity analyses first excluded participants with diabetes-range glycaemia (HbA1c ≥ 6.5% or fasting glucose ≥ 126 mg/dL) and then examined a stricter, strictly normoglycaemic subgroup (HbA1c < 5.7% and fasting glucose < 100 mg/dL) as a sensitivity analysis. Influential-observation analyses used Cook’s distance (threshold 4/n), and restricted cubic spline checks did not support meaningful non-linearity for MEQ; linear models were therefore retained. Because chronotype groups differed in age, sensitivity analyses refitted the primary models with age modelled as a restricted cubic spline (knots at the 10th, 50th, and 90th percentiles) and with a quadratic term, stratified the sample at 35 years, and formally tested age × MEQ interaction terms. Analyses were performed using SPSS version 25.0 (IBM Corp., Armonk, NY, USA) and Python, version 3.13.5 (Python Software Foundation, Wilmington, DE, USA), with statsmodels, version 0.14.6. Two-sided *p* < 0.05 was considered statistically significant for prespecified primary analyses.

## 3. Results

### 3.1. Participant Characteristics and Chronotype Distribution

The study included 282 adults, of whom 142 (50.4%) were women. The median age was 28.0 years (IQR, 23.0–40.0), median BMI was 25.3 kg/m^2^ (IQR, 22.8–29.4), and mean waist circumference was 91.4 ± 12.2 cm. Poor sleep quality (PSQI > 5) was present in 146 participants (51.8%). Chronotype distribution was evening type in 73 participants (25.9%), intermediate type in 133 (47.2%), and morning type in 76 (26.9%). Age, sex distribution, BMI, waist circumference, and sleep variables differed across chronotype groups (Table 1), supporting the use of adjusted analyses rather than unadjusted group comparisons.

### 3.2. Composite Cardiometabolic Indices by Chronotype

Evening chronotype was associated with a more adverse unadjusted cardiometabolic profile: VAI, LAP, AIP, BRI, WHtR, TyG-WC, HbA1c, and RDW-SD were higher and HDL-C was lower in the evening group, and these differences remained significant after FDR correction (Table 2). PNI differed modestly across groups but was retained only as an exploratory descriptive marker.

### 3.3. Adjusted Associations of Chronotype and Sleep Quality

Before multivariable adjustment, bivariate Spearman correlation analyses showed that both MEQ and PSQI were associated with several cardiometabolic markers, although the pattern of associations differed between the two sleep–circadian measures (Supplementary Table S1). The MEQ and PSQI scores were moderately inversely correlated (Spearman ρ = −0.378, *p* < 0.001), consistent with the view that chronotype and perceived sleep quality are related but non-identical constructs. In multivariable models mutually including MEQ and PSQI scores, higher MEQ score (greater morningness) was independently associated with lower log(VAI) and lower AIP after adjustment for age, sex, physical activity, educational status, and PSQI (BMI additionally in the AIP model), whereas PSQI score was not independently associated with either primary outcome (Table 3). Among secondary outcomes, higher MEQ score was associated with lower TyG-WC after FDR correction and showed nominal associations with BRI and WHtR; PSQI showed nominal positive associations with log(LAP) and TyG-WC that did not survive FDR correction. Multicollinearity was not evident in the main adjusted models, with all VIFs below 1.35.

**Figure 1:**
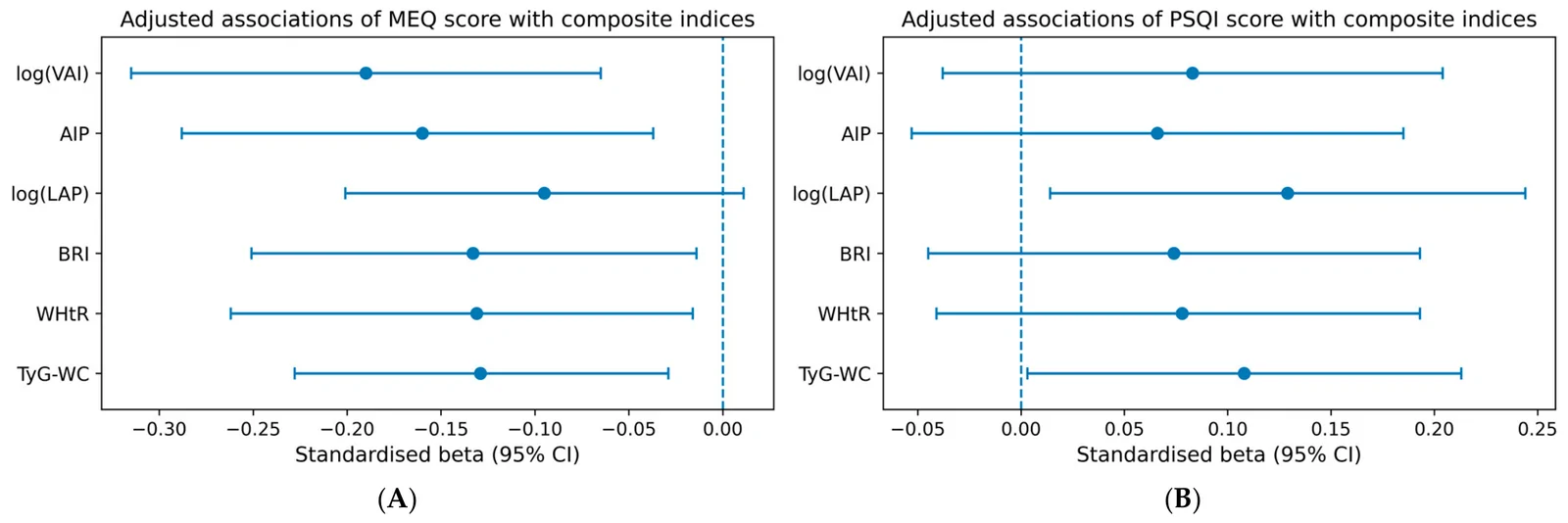
Adjusted associations of chronotype and sleep quality scores with composite cardiometabolic indices. (**A**) Standardised regression coefficients (β) and 95% confidence intervals for the associations of MEQ score with log(VAI), AIP, log(LAP), BRI, WHtR, and TyG-WC. (**B**) Standardised regression coefficients (β) and 95% confidence intervals for the corresponding associations of PSQI score with the same indices. Models were adjusted for age, sex, educational status, physical activity, and the mutually adjusted sleep variable; BMI was additionally included in the AIP model. Higher MEQ indicates greater morningness, whereas higher PSQI indicates poorer sleep quality. MEQ: Morningness–Eveningness Questionnaire; PSQI: Pittsburgh Sleep Quality Index; VAI: Visceral Adiposity Index; AIP: Atherogenic Index of Plasma; LAP: Lipid Accumulation Product; BRI: Body Roundness Index; WHtR: waist-to-height ratio; TyG-WC: triglyceride–glucose index × waist circumference; CI: confidence interval.

These adjusted associations are visually summarised in Figure 1. Greater morningness was associated with consistently lower values across the composite cardiometabolic indices, with the most robust inverse associations observed for log(VAI), AIP, and TyG-WC. In contrast, PSQI score showed weaker and less consistent adjusted associations, supporting the interpretation that chronotype and perceived sleep quality capture overlapping but non-identical dimensions of sleep–circadian health.

### 3.4. Sex-Specific Analyses

Sex × MEQ interactions were nominally significant for AIP and log(VAI), indicating a stronger inverse MEQ association in women than in men; however, after FDR correction across all tested interaction outcomes, the interaction q-values no longer met the 0.05 threshold (Table 4A). In the interaction models, mean-centring MEQ before calculating the sex × MEQ term reduced non-essential collinearity, and all VIFs remained below 2.72. Sex-stratified models were consistent with this pattern: in women, MEQ score was independently associated with both log(VAI) and AIP, whereas in men the corresponding associations were weaker and non-significant (Table 4B).

**Figure 2:**
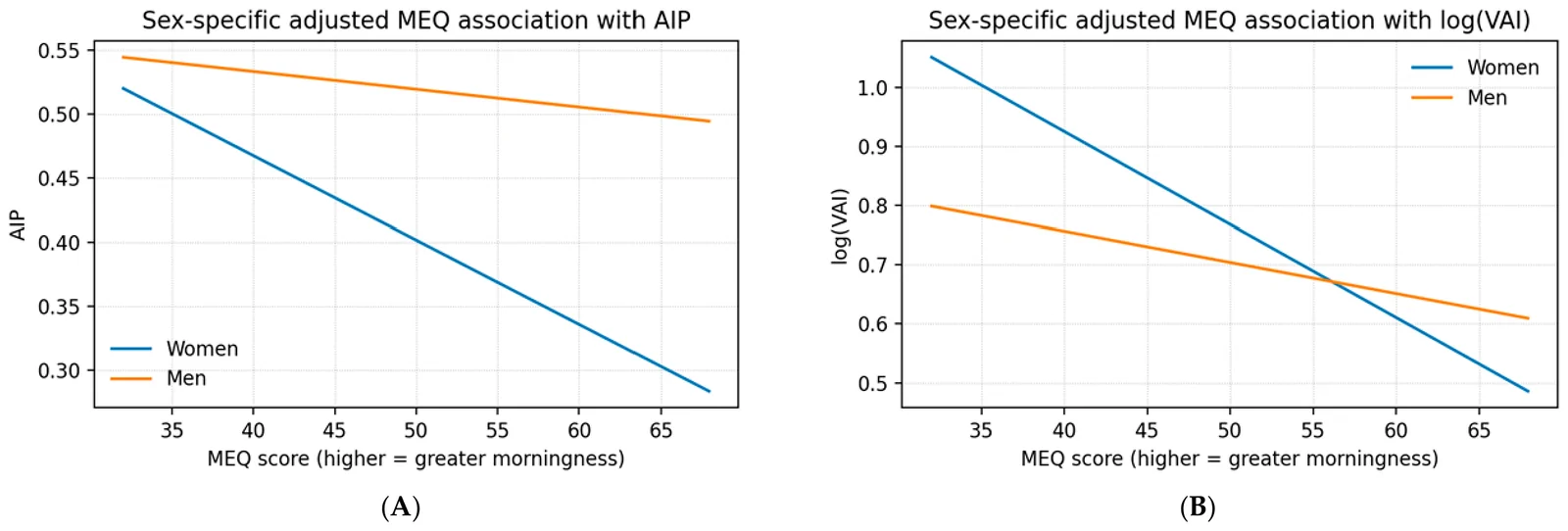
Sex-specific adjusted associations between MEQ score and the primary cardiometabolic outcomes. (**A**) Model-derived adjusted association between MEQ score and AIP in women and men. (**B**) Model-derived adjusted association between MEQ score and log(VAI) in women and men. Lines represent adjusted predictions from sex-stratified multivariable models controlling for age, educational status, physical activity, and PSQI score; BMI was additionally included in the AIP model. Higher MEQ indicates greater morningness. MEQ: Morningness–Eveningness Questionnaire; PSQI: Pittsburgh Sleep Quality Index; VAI: Visceral Adiposity Index; AIP: Atherogenic Index of Plasma; BMI: body mass index.

The sex-specific adjusted associations for the primary outcomes are illustrated in Figure 2. Visual inspection supports a steeper inverse association between MEQ score and both AIP and log(VAI) in women than in men. However, these graphical patterns should be interpreted alongside the formal interaction analyses, in which nominally significant interaction terms did not remain significant after FDR correction.

### 3.5. Glycaemic and Influential-Observation Sensitivity Analyses

Thirty-three participants had diabetes-range glycaemia on study testing (HbA1c ≥ 6.5% or fasting glucose ≥ 126 mg/dL) despite no previous diagnosis. After their exclusion (*n* = 249), the associations of MEQ score with log(VAI), AIP, and TyG-WC remained statistically significant. In the stricter normoglycaemic subgroup (HbA1c < 5.7% and fasting glucose < 100 mg/dL; *n* = 175), associations were directionally similar but attenuated and no longer significant (Table 5), indicating that the chronotype-related phenotype is not driven solely by overt diabetes-range values but may partly reflect early glycaemic–metabolic variation. Excluding observations with Cook’s distance > 4/n did not weaken the primary findings; MEQ remained associated with log(VAI) and AIP with stronger statistical evidence than in the full sample.

### 3.6. Sleep Quality

Unadjusted comparisons according to sleep-quality status are presented in Supplementary Table S2. Participants with poor sleep quality had higher visceral adiposity and atherogenic indices than good sleepers; however, once MEQ score and covariates were included, PSQI was not independently associated with the primary dependent variables. In this dataset, poor sleep quality therefore accompanied an adverse cardiometabolic profile but was not an independent correlate of VAI or AIP.

### 3.7. Age-Related Sensitivity Analyses

Because age differed markedly across chronotype groups (Table 1), further sensitivity analyses were performed. Age showed a non-linear association with log(VAI), AIP, and log(LAP) (departure from linearity *p* = 0.019, 0.024, and 0.040, respectively). Refitting the primary models with age modelled as a restricted cubic spline, or with a quadratic term, left the MEQ coefficients essentially unchanged; for log(VAI), B was −0.0102 (95% CI, −0.0165 to −0.0038; *p* = 0.002) with spline adjustment compared with −0.0096 (*p* = 0.003) in the primary model. In analyses stratified at 35 years, the association of MEQ score with log(VAI) was statistically significant in both age strata, whereas the association with AIP was significant only among participants aged 35 years or older. Associations with BRI, WHtR, and TyG-WC were significant only among participants aged under 35 years; formal age × MEQ interaction terms remained significant after FDR correction for BRI and WHtR only (q = 0.031 for both; Supplementary Table S3).

## 4. Discussion

In this cross-sectional sample of adults without previously diagnosed chronic disease, three observations emerged. First, greater eveningness was associated with a less favourable subclinical visceral-adiposity and atherogenic-lipid profile, and this association was statistically independent of perceived sleep quality. Second, perceived poor sleep quality, although associated with adverse indices in unadjusted comparisons, was not independently associated with the primary outcomes after mutual adjustment. Third, the chronotype association was numerically stronger in women, but the supporting interaction terms did not survive correction for multiple testing. All estimates are associative; given the cross-sectional design, they should not be read as evidence of causation, temporal ordering, or clinical predictive value.

The broad association between eveningness and an adverse cardiometabolic profile is already well described, and the present study is not intended to establish a novel relationship. Its contribution is instead to test that association under more discriminating conditions than are usually available: in a cohort without previously diagnosed chronic disease or regular medication use, in which early signals are less likely to be masked by established disease or treatment, with chronotype and perceived sleep quality mutually adjusted rather than conflated, with sex examined through a formal interaction term rather than stratified comparison, and with several routinely derived composite indices compared in parallel within the same cohort. By combining these features, the analysis helps clarify which component of sleep–circadian health may be most informative for subclinical cardiometabolic profiling. This framing is particularly relevant because adults without previously diagnosed chronic disease may already show metabolic heterogeneity that is not fully captured by conventional single markers. BMI reflects total body mass rather than fat distribution and cannot distinguish visceral from subcutaneous or lean compartments, so that individuals with identical BMI values may differ substantially in visceral adiposity and in insulin sensitivity; the metabolically unhealthy normal-weight phenotype, in which cardiometabolic abnormalities occur in the absence of an elevated BMI, illustrates the resulting misclassification [31]. Similarly, isolated lipid concentrations do not capture the atherogenic particle profile: the triglyceride-to-HDL-C relationship expressed by AIP corresponds to lipoprotein particle size and has been associated with cardiovascular risk beyond conventional lipid and risk-factor measurement in population-based cohorts [32]. Composite indices such as VAI, LAP, and AIP were developed precisely to recover this information from routinely available measurements [18,19], which is the rationale for their use in the present early-phenotyping framework.

The independence of the chronotype signal from perceived sleep quality is consistent with the view that the two constructs are related but non-identical [12]. This independence should nonetheless be read cautiously: because the PSQI may partly capture psychological distress, which was not separately assessed, adjustment for the PSQI cannot be equated with control for the underlying affective state. In adjusted models, MEQ score remained associated with VAI and AIP whereas PSQI did not. This finding extends prior population-level evidence linking evening preference to adverse health outcomes; in the UK Biobank, for example, evening preference was associated with morbidity and mortality after accounting for sleep-related factors [33]. Mechanistic work also supports a role for circadian misalignment in cardiometabolic regulation [2]. A reasonable interpretation is that chronotype may index the timing of behaviour relative to social demands rather than sleep quality per se; however, this remains an interpretation because the present study measured neither objective circadian phase nor behavioural mediators such as meal timing or activity rhythms. Because the design is cross-sectional, the direction of this association cannot be determined. Later chronotype may promote visceral fat accumulation through the timing of eating, activity, and sleep; alternatively, greater adiposity and its metabolic consequences may themselves shift sleep timing toward eveningness; and both may arise from shared upstream determinants, including socioeconomic position, psychological distress, and dietary pattern. These possibilities are not mutually exclusive and cannot be distinguished in the present data, and no temporal or causal ordering should be inferred.

Among the indices examined, the most consistent adjusted associations were with log(VAI), AIP, and TyG-WC. AIP is an established marker of the balance between atherogenic and anti-atherogenic lipoproteins and has been associated with coronary disease severity [21,34], so its association with eveningness is biologically coherent. Mechanisms proposed in the chrono-nutrition literature—including the metabolic consequences of late energy intake and circadian misalignment of lipid and glucose handling [3,4,35]—offer plausible explanatory pathways. However, because dietary intake and meal timing were not measured here, these pathways are necessarily speculative in the present dataset and are offered as hypotheses rather than tested mediators.

The sex-specific pattern warrants particular caution. Sex-stratified estimates were stronger in women, and the nominal sex × MEQ interactions for AIP and log(VAI) are directionally consistent with reports of sex-dependent chronotype–cardiometabolic relationships, including a higher metabolic-syndrome burden among evening-type women [13] and sex-related variation in the chronotype–obesity association [36]. Nonetheless, the interaction terms did not survive FDR correction, and the study measured neither sex-steroid status nor menstrual or menopausal phase. The data therefore generate a coherent, prespecified hypothesis of sex modification but cannot establish it; proposed hormonal or adipose-depot mechanisms remain speculative pending confirmation in adequately powered, hormonally phenotyped cohorts.

The glycaemic sensitivity analyses help to delimit the finding rather than to strengthen a causal claim. Associations persisted after excluding participants with diabetes-range glycaemia, arguing against an artefact of undiagnosed diabetes, but attenuated in the strictly normoglycaemic subgroup. The most defensible reading is that the chronotype-related phenotype is partly embedded in early glycaemic–metabolic variation—consistent with the established link between adiposity indices and dysglycaemia [37]—rather than representing an isolated adiposity signal in fully normoglycaemic individuals. Because this subgroup was substantially smaller (*n* = 175), reduced statistical precision is an alternative explanation for the loss of significance, and the two cannot be separated in the present data; the attenuation should therefore be read as a limit on the strength of the inference rather than as evidence that the association is absent in fully normoglycaemic individuals. This nuance is an interpretation supported by the data, not a demonstrated mechanism.

From a measurement perspective, the composite indices used here are convenient surrogates derived from routine data and have supportive evidence as markers of cardiometabolic risk at the population level [23,36,38,39,40]. The present results, however, do not address whether adding chronotype to such indices improves discrimination, calibration, or clinical decision-making; the modest explanatory power of the models (R^2^ ≈ 0.15–0.35) and the cross-sectional design preclude any inference about screening or risk-stratification utility. The anthropometric indices in particular appeared age-dependent, being associated with chronotype only in younger participants, which suggests that their usefulness as chronotype-related markers may not extend uniformly across the adult age range. Finally, the RDW-SD and PNI differences observed across groups are reported only as exploratory descriptive signals; the study was not designed to test inflammatory, erythropoietic, or immune–nutritional mechanisms, and these markers should not be over-interpreted.

### Limitations

Several limitations should be emphasised. The cross-sectional design prevents causal inference and cannot establish whether later chronotype precedes adverse metabolic phenotype, whether metabolic status influences sleep timing, or whether both reflect shared determinants. Reverse causation is therefore plausible, as greater adiposity may itself shift sleep timing toward eveningness. Participants were recruited from a single internal medicine outpatient clinic and should not be considered a population-based healthy sample; the most precise description is adults without previously diagnosed chronic disease or regular medication use presenting for outpatient care. The sample was restricted to adults aged 18–65 years and was young overall; the findings should therefore not be extrapolated to older adults, in whom age-related circadian phase advance and altered body composition may modify the observed associations. Chronotype groups also differed substantially in age. Although the associations were robust to non-linear adjustment for age, those with the anthropometric indices were confined to younger participants and should be regarded as age-dependent rather than uniform across the adult age range. As a single-centre study in a Turkish population, the findings may not extend to groups differing in ethnic background or cultural and environmental context, and should not be generalised beyond comparable settings.

Chronotype and sleep quality were self-reported, and objective circadian phase markers, actigraphy, polysomnography, sleep duration, and social jetlag were not assessed. Sleep duration in particular is associated with both chronotype and adiposity, so that part of the association reported here may reflect differences in habitual sleep duration rather than circadian preference as such. A broad set of psychosocial, socioeconomic, occupational, and behavioural factors—including dietary intake and meal timing, caffeine, alcohol and tobacco use, occupational category, socioeconomic position beyond educational attainment (income, employment stability, or neighbourhood environment), living and relationship circumstances, and psychological distress, as well as menstrual or menopausal status—was not measured. Because many of these are associated both with later chronotype and with greater visceral adiposity, their omission may have confounded and in part inflated the reported associations; in particular, the PSQI score may partly reflect psychological distress rather than sleep quality alone. Some participants had prediabetes- or diabetes-range laboratory values despite no previous diagnosis; sensitivity analyses addressed this, but future studies should prespecify glycaemic strata. The sample size was adequate for the primary adjusted models but limited for interaction testing, so the sex-specific findings are hypothesis-generating. Finally, composite indices are indirect and should be interpreted as surrogate phenotypic markers rather than direct measurements of visceral fat volume, insulin resistance, or lipoprotein particle size.

## 5. Conclusions

In adults without previously diagnosed chronic disease, later chronotype was associated with higher subclinical visceral adiposity and atherogenic dyslipidaemia indices independently of perceived sleep quality, and the association appeared stronger in women. These findings extend previous chronotype–cardiometabolic research by showing that the association of later chronotype with adverse routine composite indices is not fully explained by perceived sleep quality and may be more detectable in women. Because these findings are cross-sectional and the sex interaction did not survive multiple-testing correction, they are best regarded as associative and hypothesis-generating rather than as evidence of causation or clinical utility. Prospective studies incorporating objective circadian phase, objective sleep assessment, dietary timing, and broader behavioural and hormonal measures are required before chronotype assessment can be considered for early, sex-aware cardiometabolic phenotyping.

## Figures and Tables

**Table 1:** Participant characteristics by chronotype.

Variable	Evening (*n* = 73)	Intermediate (*n* = 133)	Morning (*n* = 76)	*p*
Age, years	24.0 (22.0–39.0)	26.0 (22.0–37.0)	38.0 (26.0–46.0)	<0.001 *
Women, *n* (%)	30 (41.1)	82 (61.7)	30 (39.5)	0.002 *
BMI, kg/m^2^	26.7 (24.8–30.1)	24.5 (21.8–29.1)	25.3 (23.9–28.2)	0.025 *
Waist circumference, cm	96.0 ± 11.7	88.5 ± 12.8	92.2 ± 10.2	<0.001 *
University education or above, *n* (%)	40 (54.8)	71 (53.4)	34 (44.7)	0.387
Physically active, *n* (%)	23 (31.5)	46 (34.6)	27 (35.5)	0.860
PSQI global score	8.0 (6.0–9.0)	5.0 (4.0–8.0)	4.0 (3.0–6.0)	<0.001 *
Poor sleep quality, *n* (%)	61 (83.6)	61 (45.9)	24 (31.6)	<0.001 *
MEQ score	35.0 ± 4.6	51.1 ± 4.5	63.9 ± 4.5	—

Values are presented as median (interquartile range), mean ± standard deviation, or *n* (%), as appropriate. Chronotype was classified using MEQ cut-offs: evening type, ≤41; intermediate type, 42–58; morning type, ≥59. *p*-values were calculated using the Kruskal–Wallis test, one-way ANOVA, or chi-square test, as appropriate. * *p* < 0.05 was considered statistically significant. No inferential *p*-value is reported for MEQ score because MEQ was used to define chronotype groups. BMI: body mass index; MEQ: Morningness–Eveningness Questionnaire; PSQI: Pittsburgh Sleep Quality Index.

**Table 2:** Composite adiposity, atherogenic, and exploratory markers by chronotype.

Variable	Evening (*n* = 73)	Intermediate (*n* = 133)	Morning (*n* = 76)	*p*	q
VAI	2.2 (1.7–3.0)	1.7 (1.1–2.2)	1.6 (1.0–2.1)	<0.001	<0.001
LAP	49.7 (26.5–71.1)	28.9 (18.0–52.6)	37.6 (21.2–64.2)	0.004	0.004
AIP	0.14 ± 0.20	0.00 ± 0.25	0.02 ± 0.27	<0.001	<0.001
BRI	4.8 ± 1.5	4.0 ± 1.6	4.3 ± 1.4	0.002	0.002
WHtR	0.57 ± 0.07	0.53 ± 0.08	0.54 ± 0.07	0.001	0.002
TyG-WC	828.9 ± 120.6	752.1 ± 135.5	789.0 ± 123.6	<0.001	<0.001
HbA1c, %	5.9 (5.5–6.2)	5.2 (4.9–5.5)	5.2 (4.7–5.5)	<0.001	<0.001
HDL-C, mg/dL	37.0 (36.0–38.0)	46.0 (41.0–53.0)	47.0 (40.0–54.0)	<0.001	<0.001
RDW-SD, fL	44.4 ± 2.9	42.3 ± 3.3	43.4 ± 3.7	<0.001	<0.001
PNI	48.2 (44.2–51.9)	50.8 (44.8–55.6)	49.5 (46.2–53.5)	0.037	0.037

Values are presented as median (interquartile range) or mean ± standard deviation, as appropriate. *p*-values were calculated using the Kruskal–Wallis test or one-way ANOVA, as appropriate. q-values are Benjamini–Hochberg FDR-adjusted *p*-values across the variables shown in the table. *p* < 0.05 was considered statistically significant; however, interpretation should primarily consider q-values because multiple comparisons were performed. AIP is expressed using molar lipid units. PNI was calculated as albumin (g/L) + 0.005 × total lymphocyte count (/mm^3^); it was not a prespecified cardiometabolic outcome and is shown for descriptive completeness only. VAI: Visceral Adiposity Index; LAP: Lipid Accumulation Product; AIP: Atherogenic Index of Plasma; BRI: Body Roundness Index; WHtR: waist-to-height ratio; TyG-WC: triglyceride–glucose index–waist circumference; HbA1c: glycated haemoglobin; HDL-C: high-density lipoprotein cholesterol; RDW-SD: red cell distribution width–standard deviation; PNI: Prognostic Nutritional Index.

**Table 3:** Multivariable associations of MEQ and PSQI scores with cardiometabolic indices.

Dependent Variable	MEQ B (95% CI)	MEQ β	MEQ *p*	MEQ q	PSQI B (95% CI)	PSQI β	PSQI *p*	PSQI q	R^2^
log(VAI)	−0.0096 (−0.0159, −0.0033)	−0.190	0.003	0.032	0.0141 (−0.0064, 0.0346)	0.083	0.177	0.230	0.148
AIP	−0.0035 (−0.0063, −0.0008)	−0.160	0.011	0.044	0.0049 (−0.0039, 0.0137)	0.066	0.273	0.273	0.191
log(LAP)	−0.0062 (−0.0131, 0.0007)	−0.095	0.079	0.118	0.0284 (0.0031, 0.0537)	0.129	0.028	0.056	0.257
BRI	−0.0179 (−0.0338, −0.0019)	−0.133	0.028	0.056	0.0334 (−0.0203, 0.0870)	0.074	0.223	0.243	0.223
WHtR	−0.0008 (−0.0016, −0.0001)	−0.131	0.026	0.056	0.0017 (−0.0009, 0.0042)	0.078	0.192	0.230	0.238
TyG-WC	−1.49 (−2.63, −0.34)	−0.129	0.011	0.044	4.19 (0.11, 8.28)	0.108	0.044	0.075	0.354

Each row represents a separate multivariable linear regression model with the listed cardiometabolic index as the dependent variable. Models used heteroscedasticity-consistent type 3 (HC3) robust standard errors and were adjusted for age, sex, educational status, physical activity, and the mutually adjusted sleep variable; BMI was additionally included in the AIP model. MEQ and PSQI scores were analysed as continuous variables. Higher MEQ indicates greater morningness, whereas higher PSQI indicates poorer sleep quality. B represents the unstandardised regression coefficient; β represents the standardised regression coefficient; CI, confidence interval. q-values are Benjamini–Hochberg FDR-adjusted *p*-values across the MEQ and PSQI associations shown in the table. q < 0.05 was considered statistically significant after FDR correction. VAI: Visceral Adiposity Index; AIP: Atherogenic Index of Plasma; LAP: Lipid Accumulation Product; BRI: Body Roundness Index; WHtR: waist-to-height ratio; TyG-WC: triglyceride–glucose index–waist circumference; MEQ: Morningness–Eveningness Questionnaire; PSQI: Pittsburgh Sleep Quality Index; BMI: body mass index.

**Table 4:** (**A**). Sex × MEQ interaction analyses. (**B**). Sex-stratified adjusted associations of MEQ score with primary outcomes.

(**A**)					
**Dependent Variable**	**Sex × MEQ B (95% CI)**		***p***	**q**	
log(VAI)	0.0105 (0.0006, 0.0203)		0.037	0.111	
AIP	0.0052 (0.0009, 0.0095)		0.018	0.107	
log(LAP)	0.0093 (−0.0024, 0.0211)		0.119	0.143	
BRI	−0.0257 (−0.0549, 0.0035)		0.084	0.143	
WHtR	−0.0011 (−0.0025, 0.0002)		0.100	0.143	
TyG-WC	0.12 (−2.06, 2.29)		0.916	0.916	
Each row represents a separate multivariable linear regression model with the listed cardiometabolic index as the dependent variable. The interaction term tested whether the association between MEQ score and each dependent variable differed by sex. Models were adjusted for PSQI score, age, educational status, physical activity, and BMI where applicable; BMI was included in the AIP model but not in models for indices that mathematically incorporate anthropometric components. Sex was coded as female = 0 and male = 1; therefore, positive interaction coefficients indicate attenuation of the inverse MEQ association in men relative to women. B represents the unstandardised regression coefficient; CI, confidence interval. q-values are Benjamini–Hochberg FDR-adjusted *p*-values across the interaction tests shown. q < 0.05 was considered statistically significant after FDR correction. MEQ: Morningness–Eveningness Questionnaire; VAI: Visceral Adiposity Index; AIP: Atherogenic Index of Plasma; LAP: Lipid Accumulation Product; BRI: Body Roundness Index; WHtR: waist-to-height ratio; TyG-WC: triglyceride–glucose index–waist circumference.					
(**B**)					
**Sex**	**Dependent Variable**	***n***	**MEQ B (95% CI)**	**β**	***p***
Women	log(VAI)	142	−0.0182 (−0.0264, −0.0100)	−0.349	<0.001 *
	AIP	142	−0.0073 (−0.0106, −0.0041)	−0.335	<0.001 *
Men	log(VAI)	140	−0.0041 (−0.0141, 0.0059)	−0.084	0.418
	AIP	140	−0.0013 (−0.0058, 0.0031)	−0.063	0.558
Each row represents a separate sex-stratified multivariable linear regression model with the listed cardiometabolic index as the dependent variable. Models were adjusted for PSQI score, age, educational status, and physical activity; BMI was additionally included in the AIP model. MEQ score was analysed as a continuous variable, with higher scores indicating greater morningness. B represents the unstandardised regression coefficient; β represents the standardised regression coefficient; CI, confidence interval. VAI: Visceral Adiposity Index; AIP: Atherogenic Index of Plasma; MEQ: Morningness–Eveningness Questionnaire; * *p* < 0.05 was considered statistically significant.					

**Table 5:** Glycaemic sensitivity analyses for MEQ associations.

Analytical Sample	Dependent Variable	*n*	MEQ B (95% CI)	β	*p*
Full cohort	log(VAI)	282	−0.0096 (−0.0159, −0.0033)	−0.190	0.003 *
	AIP	282	−0.0035 (−0.0063, −0.0008)	−0.160	0.011*
	TyG-WC	282	−1.49 (−2.63, −0.34)	−0.129	0.011 *
Excl. diabetes-range	log(VAI)	249	−0.0088 (−0.0156, −0.0020)	−0.171	0.011 *
	AIP	249	−0.0033 (−0.0062, −0.0004)	−0.145	0.026 *
	TyG-WC	249	−1.17 (−2.27, −0.07)	−0.105	0.038 *
Strict normoglycaemic	log(VAI)	175	−0.0068 (−0.0157, 0.0021)	−0.123	0.132
	AIP	175	−0.0026 (−0.0064, 0.0011)	−0.107	0.172
	TyG-WC	175	−0.33 (−1.58, 0.93)	−0.030	0.608

Each row represents a separate multivariable linear regression model with the listed cardiometabolic index as the dependent variable. Models were adjusted as in the main analysis: age, sex, educational status, physical activity, and PSQI score; BMI was additionally included in the AIP model. MEQ score was analysed as a continuous variable, with higher scores indicating greater morningness. The full cohort included all participants; the diabetes-range exclusion analysis excluded participants with HbA1c ≥ 6.5% or fasting glucose ≥ 126 mg/dL; the strict normoglycaemic subgroup included participants with HbA1c < 5.7% and fasting glucose < 100 mg/dL. B represents the unstandardised regression coefficient; β represents the standardised regression coefficient; CI, confidence interval. * *p* < 0.05 was considered statistically significant. VAI: Visceral Adiposity Index; AIP: Atherogenic Index of Plasma; BMI: body mass index; TyG-WC: triglyceride–glucose index–waist circumference; MEQ: Morningness–Eveningness Questionnaire; PSQI: Pittsburgh Sleep Quality Index; HbA1c: glycated haemoglobin.

## Data Availability

The data supporting the findings of this study are available from the corresponding author upon reasonable request.

## Supplementary Materials

The following supporting information can be downloaded at: https://www.mdpi.com/article/10.3390/medicina62081472/s1, Figure S1, flow of participants through the study; Table S1, Spearman correlations of MEQ and PSQI scores with cardiometabolic markers; Table S2, unadjusted comparisons of cardiometabolic markers according to sleep-quality status; Table S3, sensitivity analyses for age: associations of MEQ score with cardiometabolic indices stratified by age and formal age × MEQ interaction tests.

## Abbreviations

The following abbreviations are used in this manuscript:

AIP	Atherogenic Index of Plasma
BMI	Body Mass Index
BRI	Body Roundness Index
CI	Confidence Interval
FDR	False Discovery Rate
HC3	Heteroscedasticity-Consistent Type 3
HDL-C	High-Density Lipoprotein Cholesterol
HbA1c	Glycated Haemoglobin
IQR	Interquartile Range
LAP	Lipid Accumulation Product
MEQ	Morningness–Eveningness Questionnaire
PNI	Prognostic Nutritional Index
PSQI	Pittsburgh Sleep Quality Index
RDW-SD	Red Cell Distribution Width–Standard Deviation
SD	Standard Deviation
TG	Triglycerides
TyG-WC	Triglyceride–Glucose Index–Waist Circumference
VAI	Visceral Adiposity Index
VIF	Variance Inflation Factor
WHtR	Waist-to-Height Ratio
