# Supplementary material for: Chronotype and Atherogenic–Adiposity Indices in Adults Without Previously Diagnosed Chronic Disease: A Sex-Aware Cross-Sectional Study
Source: Medicina (Kaunas). 2026 Jul 29;62(8):1472. doi: 10.3390/medicina62081472 (PMC13514820; doi:10.3390/medicina62081472)
Supplement: Supplementary file 1 [file medicina-62-01472-s001.zip › Supplementary Figure S1-New.pdf]

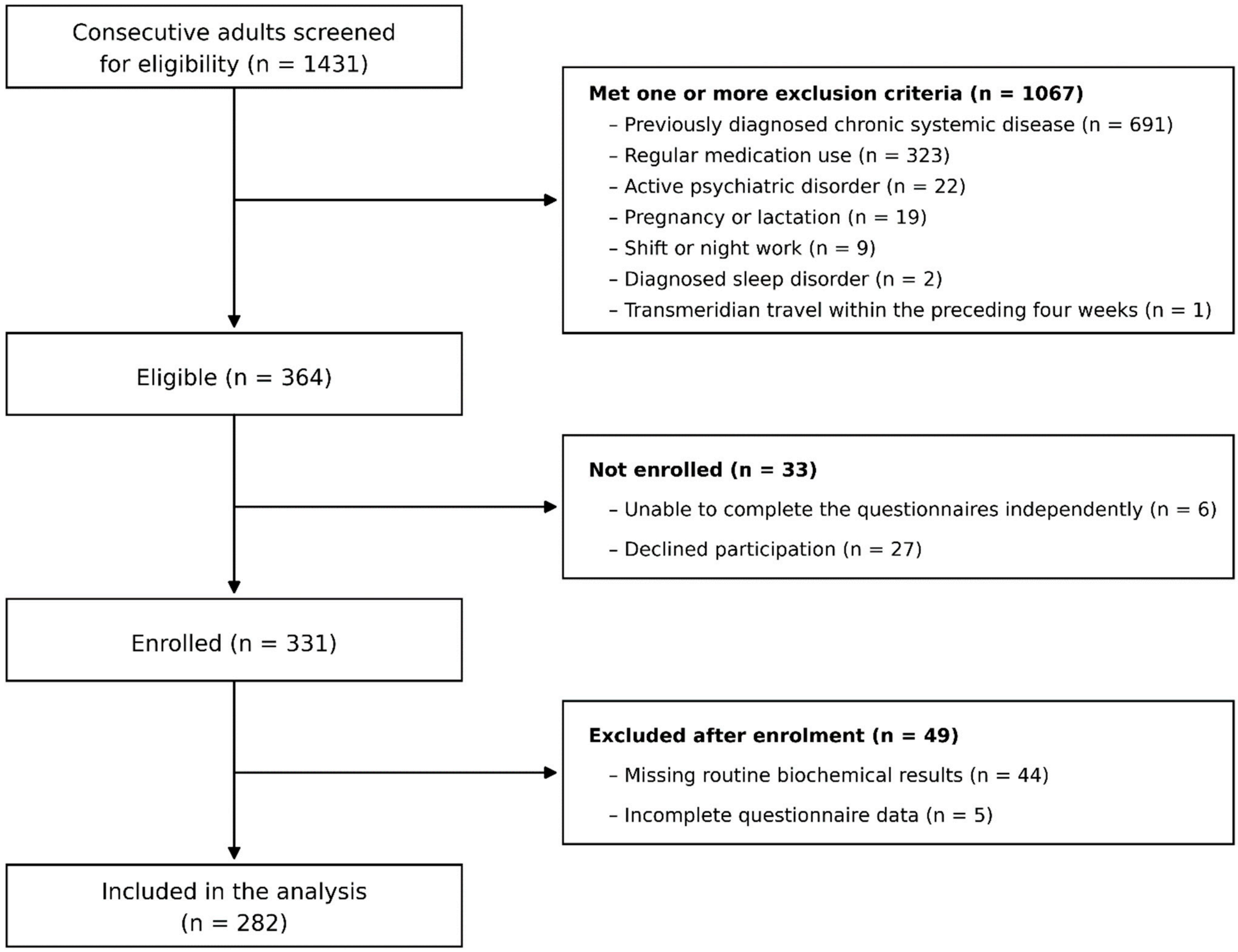

Supplementary Figure S1. Flow of participants through the study. Participants meeting more than one exclusion criterion were counted once, under the first criterion met; the categories are therefore mutually exclusive, although they overlap clinically, as most individuals excluded for regular medication use were taking treatment for a previously diagnosed chronic condition.
