# Supplementary material for: Chronotype and Atherogenic–Adiposity Indices in Adults Without Previously Diagnosed Chronic Disease: A Sex-Aware Cross-Sectional Study
Source: Medicina (Kaunas). 2026 Jul 29;62(8):1472. doi: 10.3390/medicina62081472 (PMC13514820; doi:10.3390/medicina62081472)
Supplement: Supplementary file 1 [file medicina-62-01472-s001.zip › Supplementary Table S1 Medicina.pdf]

Supplementary Table S1. Spearman correlations of PSQI and MEQ scores with cardiometabolic markers.

Score	Variable	rho	*p*	q
PSQI	VAI	0.185	0.002	0.010*
	LAP	0.168	0.005	0.017*
	WHtR	0.148	0.013	0.028*
	BRI	0.148	0.013	0.028*
	AIP	0.130	0.029	0.053
	TyG-WC	0.123	0.039	0.065
	HbA1c	0.175	0.003	0.014*
	HDL-C	−0.082	0.169	0.218
	RDW-SD	0.202	<0.001	0.005*
	Albumin	−0.141	0.018	0.036*
MEQ	VAI	−0.156	0.009	0.024*
	LAP	−0.047	0.433	0.433
	WHtR	−0.058	0.335	0.351
	BRI	−0.058	0.335	0.351
	AIP	−0.159	0.008	0.024*
	TyG-WC	−0.070	0.238	0.276
	HbA1c	−0.281	<0.001	<0.001*
	HDL-C	0.354	<0.001	<0.001*
	RDW-SD	−0.092	0.122	0.168
	Albumin	0.095	0.110	0.162

Values are Spearman rank correlation coefficients (rho) between PSQI or MEQ scores and cardiometabolic markers. q-values are Benjamini–Hochberg false-discovery-rate (FDR)-adjusted *p*-values across the correlations shown in the table. *$q < 0.05$ was considered statistically significant after FDR correction. Higher PSQI indicates poorer sleep quality, whereas higher MEQ indicates greater morningness. PSQI: Pittsburgh Sleep Quality Index; MEQ: Morningness–Eveningness Questionnaire; VAI: Visceral Adiposity Index; LAP: Lipid Accumulation Product; WHtR: waist-to-height ratio; BRI: Body Roundness Index; AIP: Atherogenic Index of Plasma; TyG-WC: triglyceride–glucose index × waist circumference; HbA1c: glycated haemoglobin; HDL-C: high-density lipoprotein cholesterol; RDW-SD: red cell distribution width–standard deviation.
