# Supplementary material for: Chronotype and Atherogenic–Adiposity Indices in Adults Without Previously Diagnosed Chronic Disease: A Sex-Aware Cross-Sectional Study
Source: Medicina (Kaunas). 2026 Jul 29;62(8):1472. doi: 10.3390/medicina62081472 (PMC13514820; doi:10.3390/medicina62081472)
Supplement: Supplementary file 1 [file medicina-62-01472-s001.zip › Supplementary Table S2 Medicina.pdf]

Supplementary Table S2. Composite indices by sleep quality.

Variable	Good sleep	Poor sleep	*p*	q
VAI	1.6 (1.1–2.1)	1.9 (1.4–2.9)	0.001	0.007
LAP	29.2 (19.6–51.5)	43.7 (24.7–68.1)	0.003	0.012
AIP	0.01 ± 0.24	0.07 ± 0.26	0.046	0.046
BRI	4.1 ± 1.5	4.5 ± 1.5	0.019	0.026
WHtR	0.53 ± 0.07	0.55 ± 0.07	0.017	0.025
TyG-WC	764.3 ± 128.9	798.4 ± 133.2	0.030	0.036
PNI	50.6 (46.2–54.5)	48.6 (43.8–53.4)	0.015	0.025
Albumin, g/L	40.0 (36.0–42.0)	38.5 (34.0–41.0)	0.009	0.022
HbA1c, %	5.3 (4.9–5.5)	5.5 (5.0–6.0)	0.006	0.019
HDL-C, mg/dL	46.0 (40.0–52.0)	42.0 (37.0–49.0)	0.011	0.022
RDW-SD, fL	42.3 ± 3.1	43.9 ± 3.6	<0.001	<0.001
BMI, kg/m^2	24.8 (22.5–28.4)	25.4 (23.5–30.1)	0.046	0.046

Values are presented as median (interquartile range) or mean ± standard deviation, as appropriate. Poor sleep quality was defined as PSQI global score >5. *p*-values were calculated using the Mann–Whitney U test or independent-samples t-test, as appropriate. q-values are Benjamini–Hochberg false-discovery-rate (FDR)-adjusted *p*-values across the variables shown in the table. q < 0.05 was considered statistically significant after FDR correction. AIP is expressed using molar lipid units. PNI was calculated as albumin (g/L) + 0.005 × total lymphocyte count (/mm^3). VAI: Visceral Adiposity Index; LAP: Lipid Accumulation Product; AIP: Atherogenic Index of Plasma; BRI: Body Roundness Index; WHtR: waist-to-height ratio; TyG-WC: triglyceride–glucose index × waist circumference; PNI: Prognostic Nutritional Index; HbA1c: glycated haemoglobin; HDL-C: high-density lipoprotein cholesterol; RDW-SD: red cell distribution width–standard deviation; BMI: body mass index; PSQI: Pittsburgh Sleep Quality Index.
