# Supplementary material for: Chronotype and Atherogenic–Adiposity Indices in Adults Without Previously Diagnosed Chronic Disease: A Sex-Aware Cross-Sectional Study
Source: Medicina (Kaunas). 2026 Jul 29;62(8):1472. doi: 10.3390/medicina62081472 (PMC13514820; doi:10.3390/medicina62081472)
Supplement: Supplementary file 1 [file medicina-62-01472-s001.zip › Supplementary Table S3 Medicina-New.pdf]

Supplementary Table S3. Sensitivity analyses for age: associations of MEQ score with cardiometabolic indices stratified by age, and formal age × MEQ interaction tests.

Index	β (<35 y, *n* = 174)	*p*	β (≥35 y, *n* = 108)	*p*	Age × MEQ *p*	Age × MEQ q
**log(VAI)**	−0.184	0.042	−0.255	0.003	0.192	0.230
**AIP**	−0.145	0.106	−0.248	0.004	0.091	0.152
**log(LAP)**	−0.151	0.055	−0.068	0.449	0.700	0.700
**BRI**	−0.186	0.011	−0.020	0.867	0.009	0.031
**WHtR**	−0.178	0.014	−0.028	0.811	0.010	0.031
**TyG-WC**	−0.190	0.006	−0.056	0.574	0.101	0.152

β denotes the standardised regression coefficient for MEQ score. Models were fitted separately in participants aged under 35 years and 35 years or older, using heteroscedasticity-consistent (HC3) robust standard errors, and were adjusted for age, sex, educational status, physical activity, and PSQI score; body mass index (BMI) was additionally included in the AIP model. Age × MEQ interaction terms were tested in the full sample with age and MEQ score mean-centred. q-values are Benjamini–Hochberg false-discovery-rate (FDR)-adjusted *p*-values across the six interaction tests shown. Higher MEQ indicates greater morningness. In models refitted with age modelled as a restricted cubic spline (knots at the 10th, 50th, and 90th percentiles) or with a quadratic age term, the MEQ coefficients were essentially unchanged relative to the primary models presented in Table 3. VAI: Visceral Adiposity Index; AIP: Atherogenic Index of Plasma; LAP: Lipid Accumulation Product; BRI: Body Roundness Index; WHtR: waist-to-height ratio; TyG-WC: Triglyceride–glucose index–waist circumference; MEQ: Morningness–Eveningness Questionnaire; PSQI: Pittsburgh Sleep Quality Index.
